# Access to and Utilization of Dental Care Services by Older Adults in Nigeria: Barriers and Facilitators

**DOI:** 10.1111/scd.70040

**Published:** 2025-05-06

**Authors:** Sunkanmi Folorunsho, Victor Ajayi, Munirat Sanmori, Medinah Suleiman, Raji Abdullateef, Abdulazeez Abdulganiyu

**Affiliations:** ^1^ Department of Sociology University of Nebraska‐Lincoln Lincoln USA; ^2^ School of Social Work University of North Carolina Chapel Hill USA; ^3^ Department of Sociology Georgia State University Georgia State University Atlanta USA; ^4^ Department of Common and Islamic Law University of Ilorin Ilorin Nigeria; ^5^ Department of Sociology University of Ilorin Ilorin Nigeria; ^6^ Brooks Insights Limited FCT Abuja Nigeria

**Keywords:** barriers and facilitators, dental care access, Nigeria, older adults, oral health

## Abstract

**Aims:**

Nigeria's rapidly aging population presents urgent challenges for the healthcare system, particularly in oral health, which remains an overlooked aspect of well‐being. While older adults are especially vulnerable to dental issues such as tooth loss, periodontal disease, and oral pain, access to care remains limited due to systemic, economic, and cultural barriers. This qualitative study explored the lived experiences of older Nigerians in accessing and utilizing dental care, with the goal of identifying key barriers, facilitators, and policy‐relevant insights.

**Methods:**

Fifteen participants aged 50–83 years were purposively selected from urban and peri‐urban areas of Ilorin, North‐Central Nigeria. Using a semi‐structured interview guide, data were collected through face‐to‐face interviews and analyzed thematically following Braun and Clarke's methodology.

**Results:**

Three overarching themes emerged: (1) barriers to accessing dental care, including high treatment costs, geographic inaccessibility, mobility challenges, and long waiting times; (2) facilitators of care, such as family support, employment‐based insurance, and culturally supportive health beliefs; and (3) perceptions of dental care, with participants recognizing its importance for nutrition, dignity, and overall quality of life. Findings reveal that dental care for older adults in Nigeria is shaped by a combination of structural neglect and individual adaptation. While participants valued preventive care, financial constraints, poor infrastructure, and a lack of geriatric‐trained professionals limited consistent access. However, social support networks and respectful provider interactions were critical enablers of care.

**Conclusions:**

This study points to the need for age‐inclusive health policies that integrate dental services into primary healthcare, expand insurance coverage, and strengthen community‐based outreach. Addressing these gaps is not only a matter of public health, but of equity, dignity, and the right to age well.

## 1 Introduction

The global population of older adults is growing at an unprecedented rate. The number of individuals aged 65 and older is expected to increase from 9.3% in 2020 to 16.0% by 2050, with over 1.5 billion older adults projected worldwide [1]. This demographic shift presents significant challenges for healthcare systems worldwide. In Nigeria, the number of individuals aged 60 and above is projected to grow from over 9 million in 2016 to 26 million by 2050 [2]. With this surge in the aging population, the healthcare needs of older adults, including oral health, have become increasingly urgent. However, as in many developing nations, providing adequate dental care for Nigeria's aging population remains a significant challenge [3].

Oral health is a vital component of overall well‐being, yet it is often overlooked in both public health discourse and individual care. Poor oral health can significantly affect essential daily functions, such as eating, speaking, and socializing, ultimately reducing quality of life [4]. Older adults are particularly vulnerable to oral health issues such as xerostomia (dry mouth), periodontal disease, tooth loss, and oral cancer [5]. These conditions have broader health implications. For example, tooth loss can lead to difficulties in chewing, altering dietary habits, and increasing the risk of malnutrition. Furthermore, poor oral health has been linked to systemic conditions such as diabetes, respiratory infections, and cardiovascular disease [6], emphasizing the interconnectedness of oral and general health outcomes.

The purpose of this qualitative study was to explore the barriers and facilitators influencing access to and utilization of dental care services by older adults in Nigeria. It also aimed to identify potential solutions and policy implications to enhance dental care delivery and utilization among older Nigerians.

### 1.1 Overview of Dental Care Services by Older Adults in Nigeria

#### 1.1.1 Patterns of Dental Visits

The uptake of dental care among older adults in Nigeria remains alarmingly low, pointing to systemic challenges within the healthcare system. Research from both urban and rural areas shows that many older adults rely on symptomatic treatments rather than preventive care [7]. Symptomatic treatment refers to medical or dental care that focuses on alleviating the symptoms of a condition, such as pain or discomfort, without addressing its underlying cause [8]. In dental care, this often involves procedures like tooth extractions to relieve severe pain or infections, rather than preventive measures such as regular cleaning, fluoride treatments, or restorative work like fillings, which aim to prevent dental problems from occurring or worsening [8]. A study conducted in Benin City, Nigeria, revealed that over 50% of older adults surveyed had never visited a dental clinic, with those who did primarily seeking care during emergencies for severe pain or infections requiring tooth extractions [9]. Preventive treatments, such as cleanings or restorations, were notably absent in their dental histories. Similarly, in Lagos, data indicate that over 70% of dental visits by older adults were for extractions, reflecting a reactive rather than proactive approach to oral healthcare [10]. This reactive approach often leads to tooth loss that could have been prevented with earlier intervention. The limited emphasis on preventive care also reflects broader gaps in awareness, accessibility, and affordability within Nigeria's dental health system. This trend starkly contrasts with practices in countries such as South Africa, where structured public health programs actively promote regular dental check‐ups for older adults, fostering a culture of preventive care [11].

#### 1.1.2 Financial Constraints and Out‐of‐Pocket Payments

Financial constraints significantly hinder dental care utilization among older Nigerians. With dental services predominantly requiring out‐of‐pocket payments, many older adults prioritize essential needs such as food and housing over oral health treatments. A study conducted in Ibadan found that high treatment costs were a primary reason for avoiding dental visits, even when experiencing severe discomfort [12]. The lack of dental insurance worsens the situation, leaving older adults without financial protection for essential services. Comprehensive treatments, such as dentures or periodontal therapy, remain inaccessible to most individuals. For example, in Lagos, only wealthier individuals or those with supportive family networks were able to afford advanced dental care services, creating significant disparities in oral health outcomes [13]. In addition to these challenges, the absence of a national policy on the welfare of older Nigerians forces many to rely on their families and other informal supports for healthcare needs, including dental care [14]. This reliance often leads to inconsistent access to necessary treatments, further exacerbating oral health disparities among the older population. Moreover, the limited availability of dental services in rural areas compounds the financial barriers faced by older adults. The shortage of dental professionals and facilities in these regions means that even those willing to seek care must often travel long distances, incurring additional costs and physical strain [15].

#### 1.1.3 Cultural Beliefs and Misconceptions

Cultural beliefs shape oral health‐seeking behaviors among older Nigerians, creating barriers to accessing professional dental care. For instance, in many Nigerian communities, tooth loss is widely viewed as a natural and inevitable part of aging. This belief discourages older adults from seeking preventive or restorative care, as they often perceive dental interventions as unnecessary. According to Esan et al. [16], in rural areas, such as parts of Benue and Ebonyi States, traditional remedies, including herbal concoctions and self‐medication, are commonly used to address oral health issues. These practices are preferred over professional interventions, further delaying proper care and increasing oral health problems [17]. Fear and mistrust of dental professionals also affect dental care utilization among older Nigerians. In Lagos, a survey revealed that nearly 40% of older adults avoided dental clinics due to a fear of pain and skepticism about the motivations of dental practitioners. Many respondents believed that dentists prioritized profit over patient care, viewing their recommendations as exploitative rather than necessary [12]. This perception is reinforced by anecdotal accounts in rural communities where negative experiences during dental visits, such as excessive pain during tooth extractions, have fostered a culture of distrust toward dental professionals [17]. In some cases, traditional cultural beliefs also associate tooth loss or oral health issues with spiritual causes, further deterring individuals from seeking professional care [18]. For example, in parts of northern Nigeria, older adults may attribute dental pain to supernatural forces or curses, opting for spiritual remedies rather than dental treatments. This belief system perpetuates a cycle of neglect and worsens oral health outcomes in these regions [15].

### 1.2 Current Study

While oral health is increasingly recognized as vital to overall well‐being, there is a significant lack of studies capturing the unique experiences and challenges faced by older Nigerians in accessing dental care. Existing research often focuses on prevalence rates, treatment outcomes, or clinical data, offering limited insight into the sociocultural, economic, and systemic barriers that influence their health‐seeking behaviors. To address these gaps, we employed a qualitative approach, which remains rare in this field. By focusing on the lived experiences of older adults, we aimed to provide a unique understanding of the factors shaping their oral health outcomes. A key motivation for this study is the absence of comprehensive research on the relationship between barriers and facilitators to accessing dental care. While some studies briefly mention cost or fear of dental procedures, few investigate how these barriers interact with cultural beliefs, systemic challenges, and social support systems. Understanding these dynamics is crucial for designing effective interventions. For example, identifying facilitators can help create practical strategies to improve access to and utilization of dental care services.

Moreover, the growing importance of addressing dental problems among older Nigerians further justifies this study. As the country's population ages, poor oral health is becoming a significant public health issue, linked to systemic conditions such as cardiovascular disease, diabetes, and malnutrition. Despite these connections, oral health often remains a low priority in both individual and policy agendas. By pointing out the specific challenges faced by older adults, this study argued for the urgent need to integrate oral health into Nigeria's broader health frameworks and resource allocation strategies. This study holds the potential to inform meaningful policy changes. Understanding the facilitators that encourage older adults to seek care can help policymakers and stakeholders leverage existing community resources more effectively. These findings could contribute to developing actionable recommendations that bridge the gap between older adults and dental care services, promoting equitable access. In addition to its practical implications, this study contributes to the limited body of research on geriatric oral health in Nigeria.

## 2 Method

### 2.1 Research Design

We employed a qualitative descriptive research design to investigate the barriers and facilitators influencing dental care utilization among older adults in Nigeria. This design is particularly suitable for studies aiming to obtain rich, participant‐driven insights into health behaviors within their sociocultural contexts [19, 20]. By emphasizing participants’ lived experiences, we sought to generate a grounded understanding that can inform health interventions and policies targeting older populations.

### 2.2 Study Setting

Our research was conducted in Ilorin, the capital of Kwara State, situated in the North‐Central geopolitical zone of Nigeria. This location serves as an important administrative and economic hub for the state and neighboring regions. Ilorin is administratively divided into three local government areas (LGAs): Ilorin‐East, Ilorin‐South, and Ilorin‐West. These LGAs collectively encompass urban, peri‐urban, and some rural settlements, representing a mix of socioeconomic and cultural demographics that enrich the diversity of the study. The population in Ilorin is predominantly Yoruba, one of Nigeria's largest ethnic groups. However, Ilorin also hosts a variety of other ethnic groups, including Hausa, Fulani, Igbo, and Nupe. This diversity is attributed to the city's historical role as a trading hub and its central location, which has facilitated interethnic integration.

### 2.3 Participant Recruitment

Participants were recruited using a purposive sampling technique designed to include older adults with diverse and relevant experiences regarding dental care access and utilization. Eligibility criteria required participants to be aged 50 years or older, reside within the study area (Ilorin), and be able to provide informed consent. Individuals with cognitive impairments, hearing difficulties, or conditions that could hinder meaningful participation were excluded.

We adopted a maximum variation sampling strategy to capture a broad spectrum of perspectives, this included individuals who had previously used dental services, those who had an unmet need for care, and those who had never accessed formal dental services. This approach aimed to reflect heterogeneity in age, gender, educational level, occupational background, and dental care history [21]. While maximum variation is a recognized principle in qualitative research, we were attentive to data saturation—defined as the point at which no new themes emerged during analysis. Saturation was assessed iteratively and reached by the 13th interview, after which two additional interviews were conducted to confirm thematic consistency [22].

Participants were recruited through healthcare workers at two major public hospitals in Ilorin. These healthcare workers introduced the study to eligible individuals attending outpatient clinics (not limited to dental care) and obtained their verbal agreement to be approached by the research team. This process was conducted in a manner that respected autonomy and voluntary participation. Potential participants were given time to ask questions and consider their involvement before providing written informed consent. No monetary incentives were offered; however, participants received refreshments and transportation support as gestures of appreciation. This ensured that participation was not coerced or unduly influenced.

A total of 30 individuals were initially identified. After confirming eligibility and willingness to participate, 15 participants were selected for interviews. The final sample consisted of 9 men and 6 women, aged between 50 and 83 years (see Table 1). Participants represented a wide range of occupational roles, including retired teachers, nurses, artisans, traders, and civil servants, as well as individuals with unspecified work histories. Educational attainment ranged from primary education to postgraduate degrees. Although this level of diversity may appear broad for a relatively small sample size, we ensured rigor by combining saturation monitoring, reflective field notes, and team discussions during data analysis to preserve depth and credibility in interpretation.

**TABLE 1:** Demographic characteristics of participants.

Participant ID	Age	Gender	Occupation/Status	Education level	Marital status	Prior dental visit
P1	70	Male	Not specified	Primary school certificate	Married	No
P2	65	Male	Retired nursing officer	Not specified	Married	Yes
P3	80	Female	Trader	National diploma	Widowed	No
P4	83	Male	Retired principal research officer	National diploma	Widowed	Yes
P5	70	Female	Not specified	Primary school certificate	Married	Yes
P6	56	Male	Retired driver	Not specified	Married	No
P7	71	Female	Tailor	Bachelor's degree	Married	Yes
P8	74	Male	Driver	Secondary school certificate	Married	Yes
P9	58	Male	Welder	Secondary school certificate	Separated	No
P10	65	Male	Retired deputy director of education	Bachelor's degree	Married	Yes
P11	70	Male	Nurse	Bachelor's degree	Married	Yes
P12	67	Female	Not specified	Primary school certificate	Married	Yes
P13	50	Female	Civil servant	Higher National Diploma	Married	Yes
P14	69	Female	Registered nurse	Master's degree	Married	Yes
P15	70	Male	Arabic teacher	Higher National Diploma	Married	Yes

### 2.4 Data Collection

We conducted semi‐structured face‐to‐face interviews using an open‐ended guide (Table 2). Interviews were held at two healthcare facilities in Ilorin. Both were located within the city but served different populations. The first, situated in the state capital, primarily catered to an urban population and functioned as a regional referral center offering specialist services. The second, located near the city's outskirts, served a more mixed population, including both urban and peri‐urban residents. Its location made it especially suitable for capturing diverse healthcare access experiences.

**TABLE 2:** Interview guide for participants.

Guiding questions	Sample follow‐up questions
How are you doing today?	Would you like to share anything before we start the interview?
2. Tell me about your general health experiences.	b. Do you face any challenges with your overall health as you age?
3. Have you ever visited a dental clinic for care?	c. If yes, what prompted you to visit? b. If no, why have you not visited a dental clinic?
4. What are your thoughts on the importance of dental care for older adults?	d. Do you think it is necessary to visit a dentist regularly? b. Why do you think some people may neglect dental care?
5. Have you experienced any challenges with accessing dental care?	e. What financial challenges have you faced? b. What about transportation or waiting times?
6. Tell me about your last dental visit (if applicable).	f. What was your experience like? b. Did you face any issues during your visit?
7. How do you think dental care can be improved in your community?	g. What specific support do you think older adults need? b. Should the government or community organizations be involved?
8. Do you think cultural or religious beliefs affect decisions about seeking dental care?	h. If yes, in what ways? b. If no, why do you think this is not an issue?
9. What are the perceptions of dental care in your community?	i. Do people seek dental care only when there is a problem? b. Are preventive measures discussed in your community?
10. Have you faced any communication or language challenges when seeking dental care?	j. Were you able to understand the healthcare providers? b. Did they explain procedures in your preferred language?
11. How do you think older adults in your community can be encouraged to seek dental care?	k. Should there be more awareness campaigns? b.—Should dental care services be subsidized or made more accessible?
12. How did you first become aware of the importance of dental care?	l. Was it through healthcare workers, family, or community programs? b. How did this knowledge influence you?
13. Is there anything more you would like to share about your experience with dental care?	m. Are there any stories or suggestions you'd like to add?

Interviews were conducted by a social worker and researcher (A.T.), a trained qualitative researcher, in either English or Yoruba, depending on the participants’ preferences. Interviews lasted 30–60 min, were audio‐recorded with consent, and transcribed verbatim. Reflexively, the research team acknowledged that conducting interviews in a healthcare setting and by a researcher with health sector affiliations could potentially influence responses. To mitigate this, we emphasized confidentiality, voluntary participation, and neutrality during interviews. Interviews were conducted in private hospital rooms to foster comfort and openness.

### 2.5 Ethical Considerations

We obtained ethical approval for the study from the Kwara State University Teaching Hospital Institutional Review Committee (KWASUTHIRC), with Approval Number: KWASUTHIRC/246/VOL.II/40. The approval was granted on October 16, 2024. Participants were fully informed about the study's objectives, assured of the confidentiality of their responses, and provided written informed consent prior to participation. The study adhered to established ethical principles, including autonomy, confidentiality, and beneficence. All interviews were conducted in private settings within the healthcare facilities to ensure that participants felt safe, respected, and comfortable while sharing their experiences.

### 2.6 Data Analysis

We conducted inductive thematic analysis, drawing from Braun and Clarke's [23] methodology. This approach allowed themes to emerge directly from participants’ narratives rather than from a pre‐existing framework, consistent with grounded theory principles. The analysis followed five stages: familiarization, open coding, theme development, review, and refinement. All transcripts were imported into NVivo 12 [24] for data management and coding. Initial coding was conducted by the first author, and the emergent codes were reviewed by the second author to enhance reliability. Any discrepancies were resolved through discussion until consensus was reached. The team met regularly to refine themes and ensure consistency in interpretation.

### 2.7 Trustworthiness

To ensure credibility, we conducted member checking not only by returning transcripts to participants for verification but also by discussing preliminary findings with five participants to validate our interpretations. Dependability was established through an audit trail, including codebooks, memos, and documentation of analytic decisions. Transferability was supported by rich contextual descriptions of setting, participants, and study process. Rather than treating subjectivity as bias, we viewed researcher reflexivity and engagement as contributing to the trustworthiness and rigor of qualitative inquiry [25, 26].

## 3 Results

Participant demographic characteristics are presented in Table 1. Based on thematic analysis, three overarching themes were identified that help explain the barriers, facilitators, and perceptions shaping older adults’ access to dental care in Ilorin, Nigeria. These are (1) Barriers to Accessing Dental Care, (2) Facilitators of Dental Care Utilization, and (3) Perceptions and Beliefs About Dental Care. Subthemes under each major category illustrate the range of participant experiences and provide insight into the practical and social realities influencing care‐seeking behavior.

### 3.1 Barriers to Accessing Dental Care

#### 3.1.1 Cost of Treatment Was a Major Deterrent

Participants repeatedly identified financial barriers as the most significant factor preventing them from accessing dental care. Many older adults are retired and rely on irregular income from family support or pension, which is often insufficient to cover medical bills. Participant 9 explained: *“I had severe pain in my teeth and knew I needed to go to the hospital, but I just couldn't go because I didn't have the money. If I spend the little I have on dental care, what will I use to eat or buy my blood pressure drugs? Sometimes you have to choose between the pain you know and the hunger you can't bear. It's not that we don't want to go—it's just that we can't afford to.”*

Participant 5 echoed this concern, sharing that she had to borrow money to complete her dental treatment and expressing frustration that older adults were not prioritized for public subsidies. *“After paying the first time, I couldn't go back for the follow‐up because the bill was too much. And I'm not working again. If the government helped people like us, like they help pregnant women or children, I believe more elders would go for dental care.”*

#### 3.1.2 Distance and Poor Availability of Clinics Discouraged Care‐Seeking

Participants reported that dental clinics were located far from their homes, especially for those living in peri‐urban or outlying areas of Ilorin. For some, the journey required multiple forms of transport and navigating poorly maintained roads. Participant 1 shared: *“The only dental clinic I know is in the big teaching hospital, and that place is far from where I live. I have to enter two taxis, and by the time I get there, I'm already tired. At my age, I can't go up and down like that again. If we had small dental centers in our communities, maybe even once a week at the primary health center, it would really help people like me.”*

Participant 12 similarly stated that the distance and stress of traveling to the facility often outweighed the perceived benefit of treatment. *“Sometimes I just cancel my plan to go because the stress of getting there is too much. Even if I have the money, the distance discourages me. It's worse for people who don't have anyone to accompany them.”*

#### 3.1.3 Transportation and Physical Challenges Limited Mobility

For participants with mobility impairments or chronic health conditions, transportation was not just about money but physical capability. Participant 14 described the dilemma of needing care but lacking physical support: *“Transport is now so expensive, and I can't even stand for too long or climb okadas [motorcycles] like I used to. If my son isn't available to take me, I just stay at home. That's why so many older people silently suffer—we want help, but there's no easy way to get it.”*

#### 3.1.4 Long Waiting Times Caused Fatigue and Frustration

Crowded facilities and delayed service were additional deterrents. Many participants expressed dissatisfaction with how long they had to wait for treatment. Participant 11 recalled: *“The day I went, I waited for hours. I arrived early, but they kept delaying us. My back was hurting, and there was nowhere to sit. I was so uncomfortable that I regretted going. After that experience, I haven't gone back. I just buy over‐the‐counter medicine when the pain starts again.”* These experiences demonstrate how structural inefficiencies in the healthcare system discourage older adults from following through with dental visits, especially when combined with physical discomfort.

#### 3.1.5 Lack of Awareness About Preventive Dental Care Contributed to Delay

Participants also highlighted a general lack of knowledge about the importance of preventive care. Most people, they noted, only sought help when the condition was already severe. Participant 3 explained: *“The only time people talk about going to the dentist is when there's serious pain or swelling. Nobody in our area talks about going to check the teeth just to be sure all is well. Even at the clinic, they don't tell us much about that. If there were community talks or programs like they do for malaria or high blood pressure, it would help people know when to go.”*

### 3.2 Facilitators of Dental Care Utilization

#### 3.2.1 Family Support Enabled Access to Care

Family members played a pivotal role in overcoming structural barriers. Many participants relied on their children or relatives to arrange transportation, pay for treatment, or provide emotional support. Participant 4 explained: *“I'm lucky that my daughter is very supportive. She took me to the hospital, helped me register, and even paid for my consultation. She waited with me the whole time. Without her, I wouldn't have been able to go. It's not every old person that has that kind of support, but for me, it made all the difference.”*

Participant 15 also described how a friend who worked at the hospital helped him navigate the system: “If not for that friend, I wouldn't have known where to go. He showed me how to get to the dental unit, what to say, and how to book. These small things matter when you're old and confused in a big hospital.”

#### 3.2.2 Public Health Campaigns and Decentralized Services Were Seen as Promising Solutions

Participants strongly advocated for government involvement to improve awareness and access. Suggestions included setting up dental outreach clinics and using media to educate older adults. Participant 5 stated: *“Let the government take dental care seriously. They should send people to communities to talk to us in our language. Use radio, churches, mosques, anywhere that people gather. If people are aware, they will act. But if they don't hear about it, they won't even know it's important.”*

#### 3.2.3 Health Insurance or Employment‐Related Coverage Facilitated Care

Those who had worked in the formal sector described fewer financial constraints due to insurance or retirement benefits. Participant 14 shared: *“When I was working, I had insurance that covered my health. Even now that I'm retired, I still get some support from my employer's scheme. That's what helped me go to the dentist without thinking too much about the money. Not everyone has that, and that's a big issue.”*

#### 3.2.4 Religious and Cultural Beliefs Encourage Health‐Seeking Behavior

Contrary to assumptions that cultural or religious values may hinder formal healthcare use, participants emphasized that their beliefs supported modern medical care. Participant 15, a devout Muslim, noted: *“Our religion teaches us to seek knowledge and health from those who know. Going to the dentist is not against our faith—it is encouraged. We are taught to be clean and to take care of our bodies. I don't think anyone avoids the hospital because of religion.”*

Participant 11 similarly explained: “We are told in church to take care of ourselves. Cleanliness, including oral hygiene, is part of our faith. Some people may not go because of fear or ignorance, not because of cultural belief.”

### 3.3 Perceptions and Beliefs About Dental Care

#### 3.3.1 Dental Care Was Viewed as Essential to General Health and Well‐Being

Participants widely recognized the link between oral health and overall wellness. They described how untreated dental problems affected their ability to eat, socialize, and feel confident. Participant 14 shared: *“Tooth pain is not a small thing. When you can't eat, you lose strength. You also avoid people because of bad breath or missing teeth. Taking care of your mouth is part of taking care of your whole body.”*

#### 3.3.2 Preventive Dental Care Was Desired, Even if Underutilized

Although most participants had not regularly accessed preventive care, they expressed a desire to do so and acknowledged its importance. Participant 12 said: *“We should be going for check‐ups just like we do for blood pressure. But many of us didn't grow up with that habit. Now we know better, but the services are not close or affordable. If it was easier, we would go*.”

#### 3.3.3 Oral Health Was Tied to Dignity and Independence

Participants emphasized how maintaining oral health allowed them to enjoy food, communicate freely, and live with dignity. Participant 13 explained: *“When I couldn't chew meat because of a bad tooth, I felt like I had lost part of myself. After the treatment, I was able to eat what I liked. That gave me joy. At this age, small things like that matter.”*

## 4 Discussion

This study explored how older adults in Ilorin, Nigeria, experience and navigate access to dental care services. Three core themes were identified: barriers to dental care, facilitators of access, and beliefs and perceptions that influence utilization. These findings provide valuable insight into the complex social, financial, and cultural realities surrounding oral healthcare for aging populations in low‐resource settings.

Although previous research has documented financial and geographic obstacles to healthcare access in Nigeria [27, 28], our findings offer a deeper understanding of how these barriers play out specifically for older adults. Participants frequently described having to choose between dental care and other pressing needs, such as medications for chronic conditions. This prioritization process, shaped by limited income, reflects how oral health is often postponed until it becomes urgent. While the participants understood the value of routine check‐ups and preventive dental care, many explained that the cost of care, including transportation, made it nearly impossible to follow through. These experiences support recent studies emphasizing how affordability remains a persistent barrier among older populations in sub‐Saharan Africa [29].

In addition to financial constraints, many participants described how distance and poor transportation infrastructure discouraged care‐seeking. Older adults with mobility limitations were especially affected. Without nearby dental facilities, many depended on multiple forms of transport or assistance from relatives. These findings build on prior work [30], yet they provide richer insight into how aging itself exacerbates access barriers. Older adults explained how fatigue, long queues, and inadequate waiting areas in public hospitals made dental visits physically and emotionally taxing.

Despite these challenges, participants described key facilitators that helped them engage with care when possible. Family support emerged as a critical factor. Adult children often provided financial support, transportation, and emotional encouragement. This reflects findings by Oyinlola [31], who observed that intergenerational caregiving plays a central role in sustaining healthcare engagement among older Nigerians. What our study adds, however, is the way this support also compensates for gaps in the public healthcare system. One participant shared that her daughter not only paid for treatment but also ensured she was seen by the right provider, pointing to how family members act as both caregivers and advocates within healthcare spaces.

The role of healthcare providers as facilitators was also noteworthy. Participants shared that respectful and patient‐centered communication from dental staff positively influenced their experience and willingness to return. These accounts align with earlier findings from Gbolahan et al. [32], who emphasized the importance of provider attitude in shaping oral health‐seeking behavior. In overstretched facilities, where institutional efficiency is lacking, simple acts of empathy and guidance became meaningful interventions in themselves.

Interestingly, religious and cultural beliefs, which are sometimes assumed to be barriers to biomedical care, were instead viewed as sources of support. Participants expressed that their religious teachings encouraged them to seek medical help and maintain cleanliness, including oral hygiene. Participants also linked oral health with broader issues of dignity, comfort, and quality of life. Maintaining the ability to chew, speak clearly, and interact socially was frequently mentioned. While these associations are well‐established in global oral health research [33], the way participants described the emotional and social consequences of untreated dental issues added a local dimension to these concepts. This affirms the idea that oral health is not only a medical issue but also a matter of daily functioning and social participation.

Finally, while this study confirms existing knowledge about systemic barriers to oral healthcare, it also contributes new insights by highlighting the lived realities of older adults who must constantly negotiate between medical necessity, financial strain, family dynamics, and cultural expectations. The narratives shared here emphasize that awareness alone is not enough to improve dental care utilization.

## 5 Solutions and Policy Implications

Improving dental care access for older adults in Nigeria requires not only acknowledging structural barriers but also implementing community‐rooted, age‐sensitive policies. The insights gained from this study offer a grounded understanding of the lived experiences of older adults and provide a foundation for practical strategies that address economic, infrastructural, and sociocultural challenges. Participants’ reflections revealed that addressing dental care disparities in older age requires integrated solutions across multiple levels of the healthcare system.

### 5.1 Integration of Oral Health Into Primary Healthcare Services

Integrating oral health into primary healthcare services represents a critical strategy for increasing equitable access to dental care, particularly in under‐resourced and peri‐urban regions. Many participants described significant structural barriers that prevented them from accessing care, such as travel distance, poor mobility, long queues, and limited transportation. As one older male participant shared: *“I have bad legs and can't stand for long. Going to the hospital just for tooth pain is not easy when you know you'll have to wait for hours.”* These experiences point to the need to decentralize oral health services by embedding them within existing primary care frameworks. For example, Community Health Extension Workers (CHEWs), who are already deeply embedded in community structures, could be trained to provide basic oral health services such as dental hygiene education, pain management, and early detection of oral diseases. One participant recommended: *“If the same health workers who come around for malaria and sugar checks can also look at our teeth, many elders will be happy. At least they won't have to travel far.”* Such integration is supported by evidence from previous studies that documented the success of CHEWs in delivering immunizations, family planning, and chronic disease monitoring [34]. Given their trusted role and familiarity with local contexts, training these health workers in oral health would not only improve reach but also reduce stigma and fear related to dental visits—another concern raised by older participants.

The National Primary Health Care Development Agency (NPHCDA) offers an already existing platform that could be adapted for this purpose. Routine programs, such as immunization days and maternal and child health outreach events, could incorporate oral screening and referrals. Experiences from states like Cross River, where mobile clinics successfully extended maternal health services to hard‐to‐reach populations [35], reinforce the feasibility of such integration. Furthermore, health campaigns focusing on nutrition, hygiene, or noncommunicable diseases can be modified to include oral health components, with older adults as a key target population.

### 5.2 Establishment of Community‐Based Dental Clinics

The creation of community‐based dental clinics emerged as a strong recommendation from study participants, many of whom viewed the lack of local dental facilities as a major barrier to seeking care. Several respondents expressed how geographic inaccessibility discouraged treatment even in cases of severe pain or visible dental deterioration. One participant stated: *“If there was a small clinic near my house, I wouldn't wait until the pain is unbearable. But when I think of the journey, I just try to manage.”*

Community‐based dental clinics can bridge this gap by bringing services directly to areas with a high concentration of older adults, reducing the need for costly or physically demanding travel. Strategically locating these clinics in community hubs, such as local health centers, marketplaces, or senior centers, would align with accessibility needs. Importantly, these clinics should be designed to accommodate physical limitations commonly associated with aging. Features such as ramps, wide entrances, shaded seating, and elderly‐friendly queue systems would make these facilities more inclusive and dignified for older patients. Participants also emphasized the need for consistent availability of affordable treatment. For example, one respondent explained: *“Sometimes when you get to the hospital, they say there's no dentist or that the tools are not working. So, we just go home again.”* In addition, to ensure functionality, clinics should be equipped with basic tools for preventive care, pain relief, and simple procedures like cleanings, extractions, and temporary fillings. These services would relieve primary hospitals of nonemergency cases while encouraging early care‐seeking, which is often delayed until the problem becomes acute.

Public–private partnerships (PPPs) offer a viable model for scaling these clinics sustainably. The *Saving One Million Lives* initiative, which brought together government, private sector, and international donors to expand access to maternal and child health services, is a testament to the power of multisector collaboration [36]. Similar PPPs can be explored to fund community dental programs, train mid‐level dental providers, and subsidize care for vulnerable populations. Additionally, civil society groups, faith‐based organizations, and community associations can help mobilize awareness and support for dental outreach. Ultimately, investing in localized oral health infrastructure will not only reduce disparities in access but also empower older adults to take proactive steps toward their overall well‐being. As one participant concluded poignantly: *“To treat our teeth like our eyes or our sugar level would make many elders feel respected. We deserve care, too.”*

### 5.3 Subsidized Dental Care Programs

Affordability emerged as one of the most critical barriers to accessing dental care among older participants, particularly for those dependent on pensions, intermittent support from children, or informal income sources. Many described postponing or altogether avoiding dental treatment due to cost concerns. One elderly participant shared candidly: *“I wanted to visit a dental clinic, but I cannot afford it. Even paracetamol is expensive these days, let alone dental care.”* This statement echoed a broader pattern across interviews, highlighting how even routine procedures such as extractions or cleanings were viewed as luxuries beyond financial reach. Participants described weighing the cost of dental visits against other pressing needs, such as food, rent, or medication for chronic illnesses like hypertension or diabetes. To address these disparities, the implementation of subsidized dental care programs tailored to older adults is essential. These programs could provide free or significantly discounted services, particularly for preventive and basic interventions such as oral hygiene counseling, extractions, and denture fittings. A participant recommended: *“Let the government help us with the costs, even if it's just cleaning or checking. It would encourage many of us to go.”*

Existing models provide a roadmap for implementation. For instance, the Lagos State Health Insurance Scheme (LSHS) includes limited dental care coverage for enrollees and has shown promise in reducing out‐of‐pocket expenses [37]. Scaling such schemes nationally and including dedicated subsidies for retirees and older adults would reduce financial strain and promote early care‐seeking behaviors.

Subsidization could be structured similarly to the Tertiary Education Trust Fund (TETFund), where earmarked levies support specific health priorities. A National Oral Health Levy, for instance, could allocate dedicated funds toward free dental outreach days for the elderly. PPPs could also bolster reach and sustainability, as seen in the Kwara State Community Health Insurance Scheme [37], which successfully expanded basic health coverage through coordinated support from state agencies, private insurers, and NGOs. *“If churches and NGOs can help distribute malaria nets, maybe they can help with toothbrushes and check‐ups too,”* one participant suggested, underscoring the potential role of civil society.

### 5.4 Inclusion of Oral Health in National Health Insurance Schemes

While subsidies offer short‐term relief, long‐term sustainability requires systemic integration of oral health into national insurance frameworks. The inclusion of dental care within the National Health Insurance Authority (NHIA) would institutionalize access and shield vulnerable populations from catastrophic out‐of‐pocket spending. Participants who had access to employer‐based or private insurance described their experiences more positively. One retired civil servant stated: *“I had insurance coverage at work, so financial constraint was not a challenge. I could go for check‐ups without worrying.”* This contrast revealed the protective role of insurance in enabling routine and preventive care, and how its absence perpetuates oral health neglect among informal workers and retirees.

Expanding the NHIA benefit package to include oral health would help democratize access to care. Services such as routine cleanings, annual check‐ups, fluoride treatments, and oral health education could be bundled into the preventive care component of the scheme. Preventive coverage not only reduces the need for more expensive treatments later but also aligns with the World Health Organization's (WHO) emphasis on integrating oral health within universal health coverage. Drawing from such efforts, NHIA could adopt a phased rollout that begins with essential services and scales over time. Moreover, embedding oral health within NHIA aligns with Nigeria's broader goals under the National Health Act and its commitment to strengthening primary health systems. The move would also reduce the financial burden currently absorbed by households, especially older adults facing multiple morbidities and limited income.

### 5.5 Expanding Access Through Mobile Clinics and Public Awareness Campaigns

Mobile dental units and targeted education campaigns are crucial for reaching older adults in remote and underserved areas. Participants emphasized that transportation barriers and a lack of awareness about available services hindered their ability to seek care. As one participant noted, “Transportation problems due to the hike in fuel prices made accessing care difficult.” Mobile clinics can provide on‐site dental screenings, basic procedures, and referrals, particularly in hard‐to‐reach communities. Public education campaigns, broadcast through radio and community meetings, can address misconceptions, promote preventive behavior, and inform older adults about available services. Successful models like the “*Keep Smiling*” campaign, which combined outreach and free care in selected Nigerian communities, highlight the potential of this dual approach to shift health‐seeking behaviors over time.

### 5.6 Geriatric Dental Care Through Workforce Development and Community Engagement

The growing number of older adults in Nigeria necessitates a workforce that is adequately trained to address age‐specific oral health needs. However, participants frequently noted that many dental professionals appeared unaware or unprepared to handle common geriatric dental conditions. These include periodontal disease, dry mouth (xerostomia), tooth wear, denture fitting issues, and difficulties in communication due to hearing loss or cognitive decline. One participant emphasized this point, stating: *“When I visited the hospital, the young dentist kept asking me to open my mouth wide like I was a child. I could barely move my jaw without pain. They don't understand our problems.”*

This points to the major gap in clinical training and the need to introduce geriatric dentistry as a formal area of specialization. Establishing certificate or diploma programs in geriatric oral care within dental schools and postgraduate institutions would prepare clinicians to offer age‐sensitive, culturally appropriate, and respectful treatment to older patients. Such training could include modules on pharmacological interactions in older age, cognitive impairments (e.g., dementia), patient communication techniques, and palliative dental care. Moreover, expanding continuing professional development (CPD) programs for in‐service practitioners would ensure that existing dentists and oral health workers are upskilled without needing to return to formal school. Successful examples of this exist in India and Brazil, where short courses on geriatric dentistry have improved provider knowledge and patient satisfaction [38].

Equally important is community engagement to raise awareness and foster trust. Participants strongly advocated for collaboration between the government and respected community figures to enhance the uptake of dental services. One older man explained: “People listen more when their religious or community leaders tell them something. If our king says go for a teeth check‐up, people will go.” This suggests that interventions embedded in community networks, such as those formed through religious institutions, elder associations, or local councils, can improve the reach and legitimacy of oral health programs. The Nigerian Red Cross's partnership with local governments during vaccination drives provides a relevant model, showing that collaboration with trusted institutions increases participation and reduces resistance to health

### 5.7 Periodic Monitoring and Evaluation

The long‐term success of oral health interventions for older adults depends heavily on robust systems for monitoring, evaluation, and learning (MEL). However, existing national health surveillance platforms such as the Nigeria Demographic and Health Survey (DHS), Multiple Indicator Cluster Survey (MICS), and the National Health Management Information System (NHMIS) do not currently include oral health indicators. This omission creates a significant blind spot in national planning and obscures the scale of unmet oral health needs, particularly among the elderly. Participants frequently voiced frustration about this invisibility. As one put it, “*They come to ask about our blood pressure and malaria, but nobody ever asks about our teeth. So how will they know we are suffering?*” To address this gap, oral health metrics should be systematically integrated into national surveys and health facility reporting tools. These could include indicators such as the number of decayed, missing, and filled teeth (DMFT), frequency of dental check‐ups, access to functional dental care, treatment satisfaction, and self‐reported oral pain. When disaggregated by age, gender, and location, such data can reveal disparities and inform targeted policy interventions. For example, understanding whether older women in rural areas face more barriers than their urban male counterparts would help guide equitable resource distribution.

## 6 Conclusion and Limitation

Despite growing recognition of the importance of aging and oral health, the needs of older adults in Nigeria continue to be overlooked in both research and policy frameworks. This study offers critical insight into how older Nigerians navigate oral health challenges amid systemic neglect, financial barriers, and social invisibility. Participants described enduring preventable pain, delaying care due to cost, and struggling to access facilities that are neither age‐friendly nor geographically accessible. These lived experiences underscore a broader pattern of inequity, where dental health remains siloed from primary care and is insufficiently integrated into public health priorities.

While the study was limited to a single region and involved a relatively small sample of 15 participants, the depth of the interviews provides a strong foundation for understanding the structural and social contours of oral health inequity among older Nigerians. Admittedly, the cross‐sectional nature of the study captures participants’ experiences at one point in time and cannot account for how oral health challenges evolve or accumulate. Nevertheless, the consistency across participants’ narratives signals systemic gaps that require urgent attention. These include the absence of dental services in rural and peri‐urban clinics, a lack of insurance coverage for routine procedures, and minimal workforce training in geriatric oral health.

Participants’ accounts also reveal that the consequences of poor oral health are not confined to the mouth. They extend to mobility, nutrition, self‐image, and social participation. When a person cannot eat comfortably, speak clearly, or smile without pain, their quality of life suffers in ways that are both physical and psychological. That many participants reported hiding their symptoms, skipping medications, or relying on home remedies further highlights how structural barriers are internalized and endured in silence. These forms of invisible suffering are especially concerning in later life, when resilience may be diminished and social support is uneven.

Efforts to address these challenges must go beyond technical fixes. It is not enough to build more clinics or purchase dental equipment. We must ask which populations remain unseen in our policies, which voices are missing from national health conversations, and which forms of pain have been normalized through decades of systemic exclusion. National health surveys, insurance schemes, and community health platforms must begin to treat oral health as foundational to overall well‐being. Programs tailored to older adults must integrate dignity, accessibility, and respect at their core.

Ultimately, this study affirms that oral health among older Nigerians is not only a clinical issue but a matter of equity and inclusion. The ability to chew, smile, and speak without discomfort should not be a privilege. It should be a basic expectation of aging with dignity. Future research must include a broader range of participants across rural, urban, and socioeconomically diverse settings and explore the long‐term impacts of oral health interventions. Until then, the silence surrounding older adults’ oral health will remain a reflection of broader societal indifference. The question is no longer whether change is needed, but whether we will choose to act on what we already know.

## Ethics Statement

We obtained ethical approval for the study from the Kwara State University Teaching Hospital (formerly known as Kwara State General Hospital) with Approval Number: KWASUTH/IRC/246/VOL.II/40 on October 16, 2024.

## Conflicts of Interest

The authors declare no conflict of interest.

## Data Availability

The data that support the findings of this study are available from the corresponding author upon reasonable request.
